# Identification of a Rare Variant in the *SRD5A2* Gene in Siblings With 46,XY Disorders of Sexual Development

**DOI:** 10.1155/crig/5546459

**Published:** 2025-10-30

**Authors:** Leena Rawal, Deepak Panwar, Ravinder Kumar, Gaurav Sharma, Sumit Jangra, Reena Nakra, Vandana Lal, Vamshi Krishna Thamtam

**Affiliations:** ^1^Department of Clinical Cytogenomics, National Reference Laboratory, Dr. Lal PathLabs Ltd., Block E, Sector 18, Rohini, New Delhi 110085, India; ^2^Department of Genomics, National Reference Laboratory, Dr. Lal PathLabs Ltd., Block E, Sector 18, Rohini, New Delhi 110085, India; ^3^Center for Individualized Medicine, Mayo Clinic, 200 First Street SW, Rochester 55905, Minnesota, USA

**Keywords:** 46,XY DSD, ambiguous genitalia, karyotyping, steroid 5α-reductase Type 2 deficiency (*SRD5A2*), whole exome sequencing

## Abstract

The *SRD5A2* gene encodes the steroid 5α-reductase-2 isozyme, which converts testosterone to dihydrotestosterone and plays a key role in sexual development and androgen physiology. Deficiency of this enzyme leads to an autosomal recessive sex-linked disorder associated with ambiguous genitalia and hypovirilization/complete feminization of external genitalia in individuals with a 46,XY karyotype. The present study emphasizes the indispensable role of molecular genetic testing in siblings (Probands 1 and 2) presented with disorders of sexual development (DSD). The biochemical, cytogenetics, and molecular testing, encompassing hormonal testing, chromosomal analysis, fluorescence in situ hybridization (FISH), and whole exome sequencing (WES), were carried out at our National Reference Laboratory. In addition, Sanger sequencing confirmed the identified variant, and bioinformatics tools were used to assess its impact on protein structure and stability, thereby assessing its pathogenic potential. The biochemical profile revealed highly elevated testosterone/dihydrotestosterone in Probands 1 and 2 as 45.4 and 43.2, respectively (biological reference range < 10). The cytogenetic analysis confirmed a 46,XY karyotype, and FISH validated the presence of the *SRY* gene. WES identified a pathogenic homozygous variant, c.737G > A(p.Arg246Gln) in the *SRD5A2* gene. Protein structure predictions and stability analyses indicated the variant's damaging effects. This study supports diagnosis through comprehensive molecular testing and highlights the significance of genetic insights in managing 46,XY DSD. This case highlights phenotypic variability in siblings with the same homozygous *SRD5A2* variant from a nonconsanguineous Indian family, underscoring the need for early genetic workup and multidisciplinary evaluation in 46,XY DSD.

## 1. Introduction

Genetic variations that occur during sexual differentiation may lead to disorders of sexual development (DSD). Determining and assigning gender in individuals with DSD is critical, especially when some individuals with a virilized brain show hypovirilization of the external genitalia, emphasizing the need for careful assessment of gender identity. DSDs are categorized into two main subgroups: 46,XY DSD and 46,XX DSD, each exhibiting diverse etiologies and presentations. Globally, the incidence of 46,XX DSD, particularly congenital adrenal hyperplasia (CAH) cases, is estimated at 1 in 14,000–15,000 live births, compared to 1 in 20,000 for 46,XY DSD. These rates vary among different ethnic groups due to variations in the occurrence of pathogenic gene variants reported in genes causing DSD [1]. During fetal development, the establishment of internal and external genitalia follows a meticulously orchestrated sequence of events, encompassing phases that are influenced by both hormones and other independent factors.

The steroid 5α-reductase-2 (*SRD5A2*) isozyme has two distinct isoforms: Type 1 and Type 2. Type 1, encoded by the *SRD5A1* gene located on chromosome 5p15, plays a role in virilization during puberty. On the other hand, the Type 2 isoform encoded by the *SRD5A2* gene, on chromosome 2p23, plays a critical role in masculinizing the fetal external genitalia. This enzyme catalyzes the irreversible transformation of 4-ene-3-oxosteroid (such as testosterone [T]) into the 5 alpha-3-oxosteroid (such as 5 alpha-dihydrotestosterone [DHT]) in the presence of NADPH. It plays a role in promoting the differentiation of male external genitalia and the prostate during fetal development. *SRD5A2* deficiency is a congenital condition impacting male sexual development. It presents as an autosomal recessive disorder characterized by deficient virilization in embryonic stages and lack of masculinization due to the inability to convert T to DHT in tissues targeted by androgens. This results in a broad spectrum of atypical external genitalia at birth and irregular masculinization during puberty [2, 3]. In individuals with 46,XY DSD, external genital variations can range from seemingly normal-appearing female or male genitalia to conditions such as hypospadias, clitoromegaly, microphallus, micropenis, enlarged clitoris, bifid scrotum, or a single urethral meatus with palpable gonads [4]. The incidence of *SRD5A2*-related DSD is reported to be highest in regions with high rates of consanguineous marriages, such as Turkey, New Guinea, and the Dominican Republic [5].

Despite extensive investigations, nearly half of the 46,XY DSD patients have unexplored genetic defects, likely due to the overlapping features between 46,XY male DSD and other DSD forms, such as partial or complete androgen insensitivity syndrome, as well as abnormalities in T synthesis. Therefore, a comprehensive diagnostic evaluation incorporating clinical, biochemical, cytogenetic, and molecular parameters is crucial for accurate gender assignment and inheritance pattern determination. Around 174 disease-causing variants in the *SRD5A2* gene have been documented leading to DSD (Human Gene Mutation Database, http://www.hgmd.cf.ac.uk/ac/index.php). This study underscores the significance of cytogenetic and molecular analyses in diagnosing 46,XY DSD in siblings from a nonconsanguineous Indian family.

## 2. Materials and Methods

### 2.1. Patients

**Figure 1:**
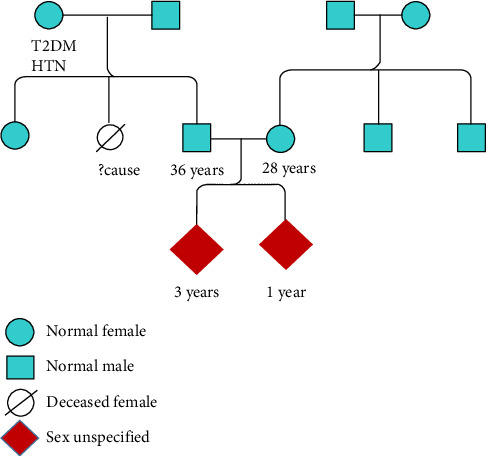
Pedigree chart of the family. The normal individuals in the family are indicated in cyan color, and the affected Probands 1 and 2 are shown in red.

Probands 1 and 2 are siblings suspected of DSD and assigned gender as females (based on the absence of characteristic male external genitalia). There is no history of consanguinity or any history of DSDs in maternal and paternal families (Figure 1). Physical examination of Proband 1 (one-year-old at presentation) with ambiguous genitalia revealed perineoscrotal hypospadias with a single perineal opening, a bifid scrotum/labia majora with palpable gonads in the lower abdomen, and a clitoral-like phallus. The transabdominal USG scan did not detect any Mullerian remnants. The Proband 2 (3 years old at presentation) was born with apparently normal-looking female external genitalia with palpable gonads in labial folds. Ultrasound examination of the pelvic region showed a blind-ending contrast-filled structure posterior to the proximal urethra, likely a Mullerian remnant. MRI of the pelvis revealed well-defined hypoechogenic structures, measuring 14 × 7 mm on the right side and 14 × 5 mm on the left side in sublabial/labioscrotal regions, likely to be testicular tissue.

### 2.2. Cytogenetic Analysis

A conventional cytogenetic chromosome analysis was performed for Probands 1 and 2. The peripheral blood lymphocytes were cultured using RPMI 1640 media, and the harvested cells were arrested at the metaphase stage by the addition of colcemid. The metaphase chromosome spreads were then stained using the GTG banding technique, and 30 metaphases per subject were analyzed and reported following the latest International System for Human Cytogenomic Nomenclature, 2020 and as described previously [6, 7].

### 2.3. Fluorescence In Situ Hybridization (FISH) Analysis

FISH was performed to confirm the presence/absence of the Y chromosome and the *SRY* gene in the interphase nuclei as well as the metaphase spreads of the lymphocytes. The manufacturer's instructions were followed using X centromere (CEP X) and *SRY*-specific (LSI SRY) probes (Vysis Inc., USA). The cells were examined using an Olympus BX63 microscope (Olympus, Tokyo, Japan) and analyzed by the Cytovision 7.0 image analysis software (Leica Biosystems, Germany) [8].

### 2.4. Whole Exome Sequencing (WES) and Variant Analysis

Genomic DNA was extracted using the Qiagen DNA Mini Kit and was quantified using a NanoDrop 2000 spectrophotometer. Followed by accurate DNA, a Qubit 3.0 fluorometer with the Qubit dsDNA High Sensitivity (HS) Assay Kit was employed. Approximately 100 ng of genomic DNA was used for the construction of exome libraries employing the Ion AmpliSeq Exome RDY panel (Thermo Fisher Scientific), according to the manufacturer's protocol. Sequencing was performed using Hi-Q chemistry on the Ion Proton platform (Thermo Fisher Scientific). Sequences were aligned against the reference genome (GRCh37/hg19) in Torrent Suite v.5.12.0 and Torrent Suite Variant Caller v.5.2.1 software, with default parameters. The annotation of the VCF file was performed using Ion Reporter v5.18 (Thermo Fisher Scientific).

### 2.5. Bioinformatics Analysis: Variant Identification and Prioritization

A comprehensive in-house bioinformatics pipeline was used for variant interpretation. WES initially yielded 38,067 variants. In the primary filtering phase, synonymous and known benign variants were excluded, and a minor allele frequency (MAF) threshold of < 0.005 was applied using databases such as gnomAD and the 1000 Genomes Project, reducing the total to 200 variants. These were further prioritized based on phenotypic correlation using Human Phenotype Ontology (HPO) terms specific to 46,XY DSD, including HP: 0000032 (ambiguous genitalia), HP: 0000062 (abnormality of the genitalia), HP: 0000056 (hypospadias), and HP: 0000078 (46,XY DSD), along with supporting literature [9, 10]. Variants were evaluated for clinical relevance, inheritance pattern, gene–disease association, and sequencing coverage. Postfiltering steps, the functional impact and pathogenicity of the 15 filtered variants were assessed using multiple *in silico* prediction tools, including SIFT4G, PolyPhen-2, and CADD-phred v1.4. Final assessment included cross-referencing with public variant databases such as ClinVar, HGMD, UniProt, and relevant DSD-related gene panels. Following phenotype–genotype correlation and database validation, the homozygous pathogenic variant in *SRD5A2* (c.737G > A; p.Arg246Gln) was selected as the primary disease-causing variant. Remaining variants were either in genes not clinically relevant to the patient's presentation or observed in heterozygous (carrier) states without supportive phenotype correlation. Variant classification was performed according to the ACMG guidelines, 2015 [11].

### 2.6. Variant Confirmation: Sanger Sequencing

The detected variant was confirmed in the siblings and their parents by employing Sanger sequencing using the BigDye Terminator v3.1 Cycle Sequencing Kit and loaded on an ABI 3500Dx automated genetic analyzer (Applied Biosystems, Thermo Fisher Scientific). Primer sequences were designed using Primer 3.0 software: forward primer, 5′-CCATCGAAATAGTCAGGCCCA-3′ and reverse primer, 5′-CAGAACGCCAGGAGACCTAC-3′.

### 2.7. Protein Structure and Stability Prediction

The three-dimensional (3D) protein model of *SRD5A2* was predicted using the Iterative Threading Assembly Refinement (I-TASSER) server [12]. Energy minimization and structural deformities of the predicted 3D structure were defined using ModRefiner Server [13]. Molecular figures were created using the program The PyMOL Molecular Graphics System (PyMOL) (Version 1.8 Schrödinger, LLC). The impact of the identified variant on the stability of protein structure was estimated using MUpro (http://mupro.proteomics.ics.uci.edu/), DynaMut (http://biosig.unimelb.edu.au/dynamut/), I-Mutant 3.0 (http://gpcr.biocomp.unibo.it/cgi/predictors/I-Mutant3.0/I-Mutant3.0.cgi), SDM (http://marid.bioc.cam.ac.uk/sdm2/), and DUET webservers, which contain Protein Data Bank (PDB) structures of query proteins to predict the Gibbs free energy (G) values. In view of the absence of functional studies, the robust in silico analysis carried out using SIFT4G, PolyPhen-2, CADD, MUpro, DynaMut, I-Mutant 3.0, and SDM was performed to predict the potential effect of p.Arg246Gln substitution.

## 3. Results

### 3.1. Biochemical Profile, Cytogenetics, and FISH

**Figure 2:**
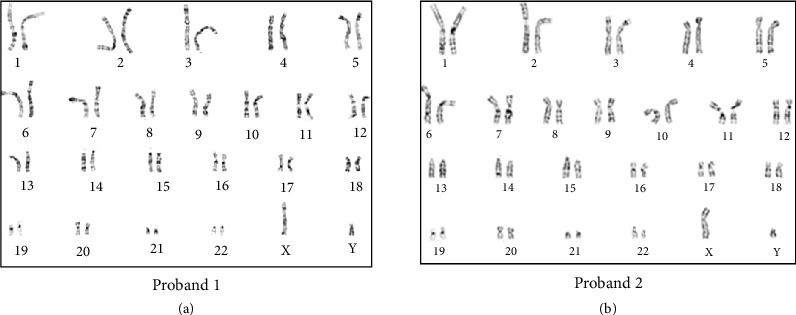
Conventional karyotyping of G-banded chromosomes. The G-banded chromosomal analysis revealed a male chromosome complement for Probands 1 (a) and 2 (b).

**Figure 3:**
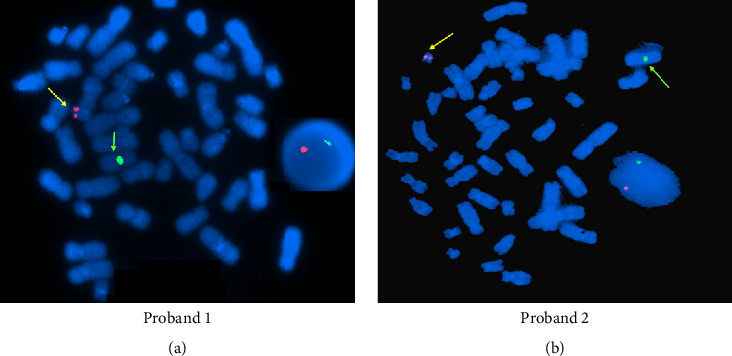
Fluorescent in situ hybridization (FISH) on metaphase chromosomes. The FISH image shows a normal X chromosome (green arrow) and the presence of the *SRY* gene (yellow arrow) on the p arm of the Y chromosome in Probands 1 (a) and 2 (b).

The serum hormone concentrations of Probands 1 and 2 have been summarized in Table 1. Both the patients (Probands 1 and 2) had a high T/DHT ratio, 45.4 and 43.2, respectively (biological reference range < 10). Initial investigations revealed normal adrenal androgen levels and normal levels of random cortisol, adrenocorticotropic hormone, and gonadotropins (Table 1). The evaluation of the G-banded chromosome preparation for Probands 1 and 2 revealed a 46,XY karyotype (Figure 2). Metaphase FISH studies confirmed the presence of the *SRY* gene on the Y chromosome in both the patients (Probands 1 and 2). Based on the phenotype from supporting tests and genotype from karyotyping, the gender for Proband 1 was reassigned as male (Figure 3). The gender reassignment decision for Proband 1 was made after thorough evaluation by a multidisciplinary team, including a pediatric endocrinologist, clinical geneticist, psychologist, and counselor. This collaborative approach ensured that the decision was based on comprehensive clinical, biochemical, and genetic findings, as well as ethical and psychosocial considerations. Written informed consent was obtained from the parents following genetic counseling. A long-term management plan has been initiated, which includes ongoing psychological support and pubertal monitoring, with provisions to re-evaluate gender identity and clinical interventions as the child matures.

On the other hand, Proband 2 is being raised as female and will be re-evaluated after a few years (at pubertal age) (Figure 3). A detailed re-evaluation is planned at the onset of puberty, which will include assessment of secondary sexual characteristics, hormonal profiling (including T, DHT, LH, and FSH), and pelvic imaging to evaluate internal reproductive structures. In addition, psychosocial evaluation and gender identity assessment will be part of the long-term management plan. It is important to acknowledge that delayed gender assignment may pose psychological and ethical challenges, including identity uncertainty, social adaptation difficulties, and decision-making stress for both the patient and family. These aspects highlight the importance of individualized, longitudinal care and continued involvement of endocrinology, genetics, and mental health specialists throughout adolescence.

### 3.2. WES Identification of the *SRD5A2* Variant

**Figure 4:**
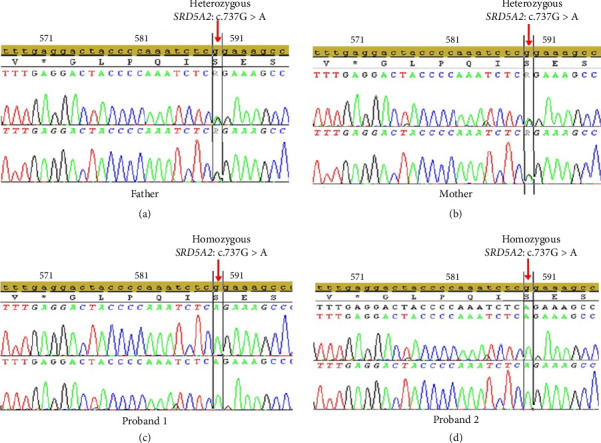
Sanger validation of the *SRD5A2* variant in the family. Sequencing chromatogram showing the variant in the *SRD5A2* gene in father (a) and mother (b), confirming the heterozygous carrier status and showing that they are normal. Affected Proband 1 (c) and Proband 2 (d), showing the homozygous state of c.737G > A variant in the *SRD5A2* gene, which was inherited from both father and mother.

Among the 15 shortlisted variants, a homozygous pathogenic variant in *SRD5A2* (c.737G > A; p.Arg246Gln) was identified through exome sequencing. This variant was predicted to be deleterious by multiple in silico tools: SIFT4G (score: 0.0), PolyPhen-2 (score: 0.999), and CADD (score: 25.5). Sanger sequencing confirmed the homozygous state of the variant in both Proband 1 and Proband 2, while both parents were found to be heterozygous carriers (Figure 4). The remaining variants were either located in genes unrelated to the clinical phenotype, predicted to be benign, or observed in carrier states and were therefore not considered clinically significant.

### 3.3. Bioinformatics: Prediction of Protein Structure and Stability Analysis

**Figure 5:**
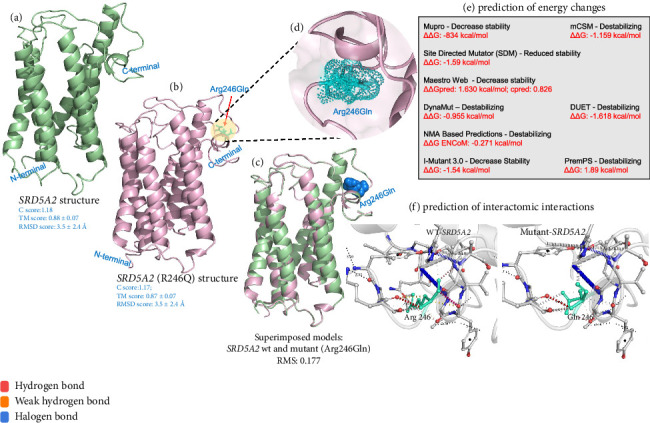
3D structure of wild-type (WT) and variant (R246Q) *SRD5A2* protein. Tertiary structures of both the WT (a) and variant, R246Q (b) are shown, with superimposed amino acid residues of the WT and variant in blue (c). A closer view of the variant, R246Q, is highlighted in light green (d). ΔG prediction outcome using different methods (e). Close view of interatomic interactions, wild-type, and mutant residues are colored in light green and are also represented as sticks alongside the surrounding residues which are involved in interactions (f). Superimposition was made, and all structures were viewed in PyMOL software.

The wild-type (WT) *SRD5A2* protein and the mutant protein structure carrying the variant R246Q were predicted by the I-TASSER web server to have the c scores of 1.18 and 1.17, respectively (Figures 5(a) and 5(b)). These scores reflect the high quality of the predicted model and the accurate folding of polypeptide chains in *SRD5A2*. The stereochemical check by Ramachandran plots of the full-length *SRD5A2* model depicted that 98.1% and 98.5% of amino acid residues in the WT and mutant forms of R246Q protein, respectively, are in the favorable region, suggesting the good stereochemical quality of the predicted structure. Superimposition of *SRD5A2* 3D structures (the native and mutant proteins) showed the changes in volume and residue score of mutant R246 (0.177 Å) compared to the native Q246 residue of *SRD5A2.* The potential effect of the R246Q variant on the tertiary structural features was explored, and we implemented a full 3D modeling structure of the *SRD5A2* protein. Computational modeling indicated that the R246Q substitution had a highly destabilizing effect, resulting in a calculated free energy difference (ΔΔG), compared to the WT protein (Figures 5(c), 5(d), 5(e), 5(f)). Energy calculation from various tools indicated that R246Q was a highly destabilizing variant. This confirms the in silico prediction of the mutant protein being “damaging” in effect.

Although in vitro enzymatic assays could not be performed to assess the functional impact of the p.Arg246Gln variant, this specific substitution is already well-established as pathogenic, with multiple independent submissions to ClinVar and previously reported in affected individuals [5]. Furthermore, application of the comprehensive panel of in silico prediction tools, including SIFT4G, PolyPhen-2, CADD, MUpro, DynaMut, I-Mutant 3.0, and SDM, predicted the deleterious and destabilizing effect on protein structure.

## 4. Discussion

Human sexual differentiation and development are highly complex processes set into motion as gonads begin developing into either ovaries or testicles during the early fetal development process, while the later development of the reproductive organs and genitalia takes place during puberty [14]. Any genetic variation during sexual differentiation may lead to “DSD.” The *SRD5A2* gene is crucial for the synthesis of DHT, which is a potent androgen responsible for the development of male secondary sexual characteristics and differentiation of male external genitalia during embryogenesis [15]. Variants in the *SRD5A2* gene cause the 5α-reductase 2 deficiency associated with DSD [16]. The majority of 46,XY DSD individuals are homozygotes and report a high degree of consanguinity; however, this condition also exists in compound heterozygotes. A vast literature survey on the mutations reported in the *SRD5A2* gene indicated that approximately 60% (150/250) were homozygous and 40% (100/250) were compound heterozygous variants. These data are comparatively less than that which was previously published, involving 55 patients, of whom 29.9% had a compound heterozygous mutation, including the V89L polymorphism, and 69.1% were homozygous [17]. There are five exons in the *SRD5A2* gene, and Exons 1 and 4 account for 33% and 19% of all variants, respectively [18, 19]. In the well-researched *SRD5A2* gene polymorphism, valine is substituted for leucine at position 89 (V89L) as a result of an exchange of guanine to cytosine in Exon 1 [19].

Genetic diagnosis plays a crucial role in the management of DSD. In the present study, Probands 1 and 2 were karyotyped as 46,XY, and exome sequencing detected a pathogenic variant, c.737G > A; p.Arg246Gln, in the *SRD5A2* gene, which leads to 5α-reductase deficiency. Sanger sequencing confirmed that Proband 2 has the same variant in the homozygous state, and that both parents are carriers of this variant. Although being uncommon in the Indian population, this variant can be found in population databases (rs9332967, gnomAD 0.1%), and ClinVar reports that it is pathogenic. In addition, the markedly elevated T-to-DHT (T/DHT) ratios observed in both siblings provide strong biochemical support for the diagnosis of 5α-reductase Type 2 deficiency, consistent with the genetic findings. Phenotypic manifestations seen in patients with this variant are the development of male pseudohermaphroditism and the requirement for surgical intervention in cases of hypospadias, which further support the variant's pathogenicity [20–26].

The p.Arg246Gln variant has been reported in various populations, including a study that identified it in two unrelated Indian families, suggesting a possible founder effect in the population [20]. The p.Arg246Gln variant has been found in several unrelated families, which suggests that it may recur and have clinical significance when combined with 5α-reductase 2 deficiency [21]. Previous large-scale studies, such as Gui et al., have provided comprehensive genotype–phenotype correlations for *SRD5A2* deficiency in Chinese cohorts, highlighting both regional variation and recurrent variants such as p.Arg246Gln [22]. However, most reported cases arise from consanguineous backgrounds and exhibit relatively consistent clinical presentations. Similarly, Sharma et al. identified p.Arg246Gln among deleterious variants in infertile Indian males but without detailed phenotypic characterization or family-based analysis [27]. In addition, published studies on *SRD5A2*-related DSD have relied either on cytogenetic analysis or targeted next-generation sequencing (NGS)–based approaches, often reporting cases in consanguineous families with limited integration of biochemical and structural data [16, 17, 20].

In contrast, our report describes a comprehensive diagnostic framework that includes hormonal profiling, cytogenetics, WES, Sanger validation, and in-depth protein modeling in two siblings from a nonconsanguineous Indian family, both homozygous for the p.Arg246Gln variant, yet exhibiting marked phenotypic divergence and differing gender assignment. This observation underscores the clinical variability and incomplete genotype–phenotype correlation associated with this variant and points to the likely influence of additional genetic or environmental factors. Our findings not only reinforce the pathogenicity of the p.Arg246Gln variant, previously reported in isolated cases [20–23], but also provide novel evidence of familial phenotypic variability, underscoring the need for a multidisciplinary approach in suspected DSD cases even when standard cytogenetic results appear normal. Thus, the identification and structural validation of the rare p.Arg246Gln variant in two affected siblings emphasizes the need for early and integrated genetic workups in DSD cases for critical clinical decision-making.

Both parents were confirmed to be heterozygous carriers of the variant but are phenotypically unaffected, with no reported history of infertility, DSD features, or hormonal abnormalities. This is consistent with previous reports that carriers of pathogenic *SRD5A2* variants are typically asymptomatic [22]. Although extended family members were not available for genetic testing, the inheritance pattern observed in this nuclear family supports autosomal recessive transmission. Moreover, both siblings carrying the same homozygous pathogenic variant (p.Arg246Gln) were presented with distinct phenotypes, Proband 1 with overt genital ambiguity requiring gender reassignment to male, and Proband 2 with apparently female external genitalia, who is currently being raised female. This phenotypic divergence, despite identical genotypes, underscores the potential involvement of additional modifying factors, including differences in prenatal androgen exposure, tissue-level androgen sensitivity, timing of diagnosis, or other genetic or epigenetic influences. Such intrafamilial phenotypic variability has also been observed in previous reports of *SRD5A2* deficiency and reflects the clinical complexity and incomplete genotype–phenotype correlation often seen in 46,XY DSD [22]. This case reinforces the need for personalized, multidisciplinary evaluation and long-term follow-up in DSD management.

Recent advancements in gene sequencing technology, especially through the use of exome sequencing assays, have demonstrated the effectiveness of an NGS-based panel approach as a valuable initial tool for diagnosing DSDs when other genetic testing results are inconclusive [28, 29]. The application of DHT gel has been reported to be beneficial over T by promoting enlargement of the penis and glands before any eventual surgery [30]. In the present study, topical DHT treatment has been advised to Proband 1. T replacement therapy is not the obvious choice as the testicular function is mostly retained during puberty in male patients. However, high doses of intramuscular T (e.g., T cypionate 50–400 mg twice a week) or DHT gel (e.g., 5–10 mg/day) are advised to improve body hair and penile length. Usually, an increase in the penile length is obtained after 6 months of high-dose treatment, but without reaching a normal length [31, 32].

## 5. Conclusions

The deficiency of the *SRD5A2* isozyme leads to ambiguous external genitalia with a variable phenotypic spectrum. Gender assignment is difficult when a patient with a 46,XY DSD presents with ambiguous genitalia. An explicit diagnosis and gender assessment prove effective in assigning gender to DSD patients with an identifiable cause. Psychosexual development, influenced by genetic status, pre/postnatal androgen exposure, sex chromosomal genes, sociocultural elements, and family dynamics, plays a vital role in the dynamic process of forming sexual identity. The present study strongly emphasizes the role of genetic diagnosis when combined with hormonal profile evaluation, molecular cytogenetics and exome sequencing, and personalized management strategies for affected 46,XY DSD patients.

## Figures and Tables

**Table 1:** Basal hormone profile of the patients.

Parameter tested	Proband 1	Proband 2	Biological reference
Testosterone (T)	236 ng/dL	212 ng/dL	Male: < 14–37 ng/dl Female: < 8.9–25 ng/dL
Testosterone (free)	0.84 ng/dL	0.83 ng/dL	< 1.3 ng/dL
Cortisol	5.73 μg/dL	9.55 μg/dL	3–15 μg/dL
Dihydrotestosterone (DHT)	5.19 ng/dL	4.9 ng/dL	2.8 ng/dL
Follicle-stimulating hormone (FSH)	4.33 mlU/mL	3.41 mlU/mL	0.26–3 mIU/mL
Luteinizing hormone (LH)	0.33 mlU/mL	0.19 mlU/mL	< 0.02–0.30 mIU/mL
Thyroid-stimulating hormone (TSH)	3.819 ulU/mL	5.692 ulU/mL	0.700–6.400 ulU/mL
Testosterone II (TSTII)	< 7 ng/dL	< 7 ng/dL	< 0.24 ng/dL
17α-Ethinylestradiol (Ee2)	15.60 pg/mL	19.08 pg/mL	Male: 3–10 pg/ml Female: 5–10 pg/mL
Beta human chorionic gonadotropin (B-HCG)	< 1.20 mlU/mL	< 1.20 mlU/mL	< 1.0 mIU/mL
Testosterone/Dihydrotestosterone ratio (T/DHT) ratio	45.4	43.2	< 10

## Data Availability

The variant identified in this study (*SRD5A2*: c.737G > A; p.Arg246Gln) is publicly available and well-documented in ClinVar. Raw whole exome sequencing data are not publicly archived due to institutional data sharing policies and patient privacy considerations. Relevant data may be furnished upon request to the corresponding author subject to institutional and ethical guidelines.
